# Trends in Survival of Patients with Primary Gastric Diffuse Large B-Cell Lymphoma: An Analysis of 7051 Cases in the SEER Database

**DOI:** 10.1155/2018/7473935

**Published:** 2018-10-16

**Authors:** Pan-pan Liu, Yi Xia, Xi-wen Bi, Yu Wang, Peng Sun, Hang Yang, Zhi-ming Li, Wen-qi Jiang

**Affiliations:** ^1^Department of Medical Oncology, Sun Yat-sen University Cancer Center, 651 Dong Feng East Road, Guangzhou 510060, China; ^2^State Key Laboratory of Oncology in South China, Guangzhou 510060, China; ^3^Collaborative Innovation Center for Cancer Medicine, Guangzhou, Guangdong 510060, China

## Abstract

Treatment modalities for primary gastric diffuse large B-cell lymphoma (PG-DLBCL) have changed significantly during the past decades. However, limited information on the trends of clinical outcome of PG-DLBCL patients has been reported. Here, we conducted a retrospective analysis using the Surveillance, Epidemiology, and End Results (SEER) database to compare the survival trends of PG-DLBCL patients from 1973 to 2014. Patients were divided into 2 eras based on the year of diagnosis in relation to immunotherapy with the anti-CD20 antibody rituximab that was approved in 1997 and became a widely used drug in 2000. There was a significant improvement in survival among PG-DLBCL patients diagnosed in the 2001–2014 era (*n* = 4186) compared to patients diagnosed in the 1973–2000 era (*n* = 2865), with the 5-year overall survival rates of 53% and 47%, respectively (*p* = 0.001). Multivariable analysis revealed that the 2001–2014 era (HR = 0.892, *p* = 0.001) was associated with lower mortality and that patients of older age, Black race, advanced stage, and male gender were associated with poor prognosis. Although outcome of PG-DLBCL has significantly improved over time, more effective therapies are needed for older patients to further improve their survival.

## 1. Introduction

Primary gastric diffuse large B-cell lymphoma (PG-DLBCL) is the most common extranodal non-Hodgkin lymphomas (NHLs) [1]. Patients with this type of lymphoma tend to present at limited stage and have a relative favorable prognosis [2, 3]. Early studies favored radical gastrectomy as the first option in the management of PG-DLBCL as its role in the diagnosis, staging, and treatment for this disease [4–6]. However, with the recent advancement in diagnostic technique, the availability of more aggressive chemotherapy regimens, and the concern on the complications caused by gastric resection, surgery has been replaced by chemotherapy and radiotherapy in the treatment of PG-DLBCL. Several investigators even suggested that surgery should be omitted as they found the overall survival (OS) of the nonsurgical group was not inferior to that of the surgical group [1, 3, 7]. Their observations suggest that chemotherapy in combination with or without radiation may be preferred.

The anti-CD20 antibody rituximab was tested for the management of B-cell lymphomas in late 1990s and approved by the FDA in November 1997. The addition of rituximab to chemotherapy regimens such as CHOP has improved the outcome of many subtypes of B-cell NHL patients, yielding 10% to 30% survival benefits. However, it is unclear if rituximab could improve the OS of PG-DLBCL patients, as the controversial results from different single-institution studies.

Although significant changes in PG-DLBCL treatment strategies, including a decrease in surgery, availability of more intensive chemotherapy regimens, and application of rituximab, have occurred in the past two decades, the impact of these changes on the survival of patients remains to be elucidated. Using the Surveillance, Epidemiology, and End Results (SEER) database, this study aimed to compare the changes in clinical outcome of PG-DLBCL during the past two eras (1973–2000 and 2001–2014) in the United States, to identify which subgroup in terms of sex, race, stage, and age might be most affected, and to evaluate the difference of outcome reported in published literature in relation to the findings from this study.

## 2. Methods

### 2.1. Data Source

The source of data for this study was from the Surveillance, Epidemiology, and End Results (SEER) database of the National Cancer Institute in the United States. SEER is a program that collects and publishes cancer incidence, treatment, and survival data from population-based cancer registries, representing approximately 28% of the US population [8]. The 18 registries in SEER-18 include approximately 25% of White population, 26% of Black population, 38% of Hispanic population, 44% of American Indians and Alaska (A/PI) population, 50% of Asians, and 67% of Hawaiian/Pacific Islanders [8]. These 18 SEER registries include Atlanta, Detroit, Greater California, Greater Georgia, Hawaii, Iowa, Kentucky, Los Angeles, New Mexico, New Jersey, Rural Georgia, Connecticut, San Francisco-Oakland, Seattle-Puget Sound, San Jose-Monterey, the Alaska Native Tumor Registry, Louisiana, and Utah.

### 2.2. Study Cohort

The SEER database uses the third edition of the International Classification of Disease for Oncology (ICD-O-3) to classify cancer histology and topography. Patients with PG-DLBCL in this study were identified using ICD-O-3 codes for histology (9680 diffuse large B-cell lymphoma [DLBCL], NOS and 9684, malig. lymphoma, large B, diffuse, and immunoblastic) and anatomically located in the stomach (ICD-O-3 topography code: C16).

For this study, we included patients with PG-DLBCL diagnosed between 1973 and 2014. We directly extracted PG-DLBCL information including year of diagnosis, age at diagnosis, race/ethnicity, clinical stage, sex, directed surgery, radiation recode, survival time, and vital status using the SEER^∗^Stat software. The patients were divided into two era groups based on year of diagnosis, 1973–2000 or 2001–2014. The recent era was expected to reflect the decreased use of surgery, application of much more intense chemotherapy regimens, availability of rituximab, and advancement in supportive care. The impact of era on survival was further stratified by age (<60 years and ≥60 years), race (White, Black, and other), gender (males and females), and disease stage (limited, Ann Arbor I and II, advanced, and Ann Arbor III and IV). Since the stage information was only available after 1983 for DLBCL, the impact of era on survival based on stage is therefore restricted to 2 time periods: 1983–2000 and 2001–2014. The variables of era of the diagnosis, age, race, stage, and gender were subjected to both univariate and multivariate analyses to assess their prognostic value on survival. Only the cases with known race and stage information were included in the multivariate analysis.

### 2.3. Statistical Analysis

Statistical analysis was performed using the SEER^∗^Stat 8.3.5 and Statistical Package for Social Sciences (SPSS) 20.0 software (IBM Corporation, Armonk, NY, USA). Kaplan-Meier survival curves were plotted and log-rank test was used to compare the survival difference. Multivariate analysis using Cox proportional hazards models was used to determine the impact of era of diagnosis, race, age, gender, and disease stage on survival. A *p* value < 0.05 was considered as statistically significant f.

## 3. Results

### 3.1. Patient Characteristics and Treatment Trends

From 1973 to 2014, a total of 7051 PG-DLBCL patients were registered in the SEER database. Of those, 2865 cases were diagnosed during 1973–2000 period and 4186 cases were diagnosed during 2001–2014 period. The patient characteristics of the two eras are shown in Table 1. The PG-DLBCL cases analyzed in this study contained 3944 (55.9%) males and 3107 (44.1%) females. The majority of patients (5692, 80.7%) were White, 528 (7.5%) were Black, and 793 (11.2%) were other races. Staging information was only available for patients diagnosed after 1983. Among patients with known disease stage, 4184 cases (59.3%) were early-stage (defined as Ann Arbor stages I and II) and 1962 cases (27.8%) were advanced-stage (defined as Ann Arbor stages III and IV) PG-DLBCL.

**Figure 1:**
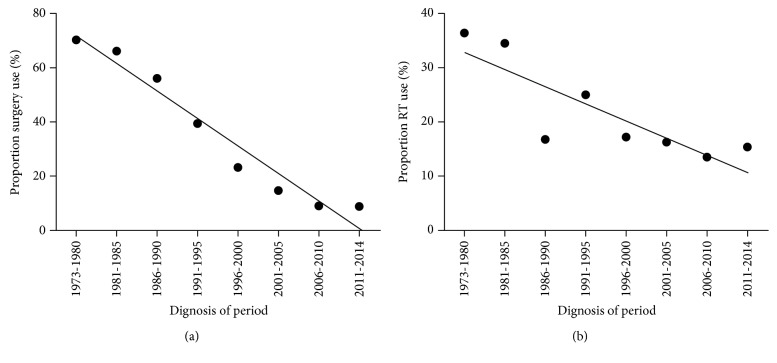
Treatment trend showing the decreased use of surgery over time (a) and decreased use of radiation (RT) over time (b) for clinical management of PG-DLBCL patients.

Analysis of the patient treatment information available in the SEER database revealed noticeable changes in the treatment strategies for PG-DLBCL during the past several decades. The use of surgery has declined from70.3% in the period of 1973–1980 to under 10% in the period of 2011–2014, while radiotherapy decreased from 36.4% in the period of 1973–1980 to less than 20% in the period of 2011–2014 (Figure 1). The combination of chemotherapy and immunotherapy has become the mainstream treatment for PG-DLBCL in the recent years.

### 3.2. Clinical Outcomes

**Figure 2:**
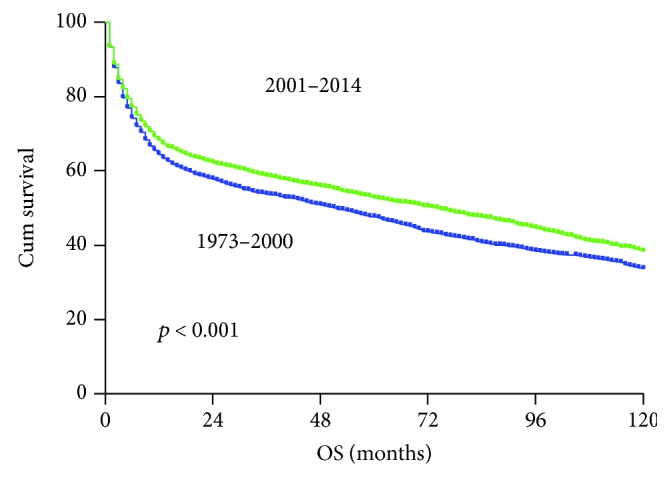
Comparison of overall survival of PG-DLBCL patients diagnosed in the 1973–2000 era (blue color) and in the 2001–2014 era (green color).

The 5-year overall survival (OS) for the whole PG-DLBCL population was 51%. As shown in Figure 2, the 5-year OS of patients diagnosed in the 2001–2014 era was 53%, representing a significant improvement from that of the 1973–2000 era (47%, *p* = 0.001).

**Figure 3:**
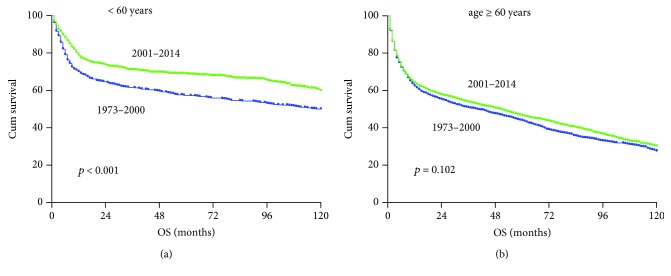
Changes in overall survival for PG-DLBCL patients diagnosed in the 1973–2000 era (blue color) and in the 2001–2014 era (green color). (a) Age < 60 years and (b) age ≥ 60 years.

Age seems to have a major impact on the patient survival irrespective of era. Improvements in survival rates across the two eras were observed only in patients under 60 years of age, with 5-year OS improved from 56% to 68% (Figure 3(a)). No improvement of overall survival was observed in patients aged 60 and older (Figure 3(b)).

**Figure 4:**
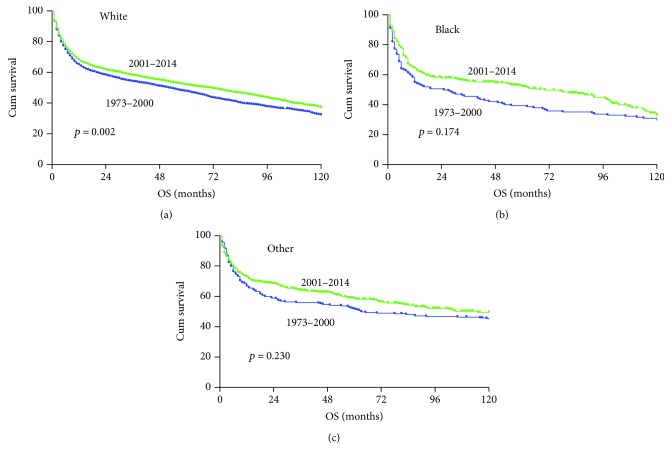
Changes in overall survival for PG-DLBCL patients of different racial groups diagnosed in the 1973–2000 era (blue color) and 2001–2014 era (green color).

**Figure 5:**
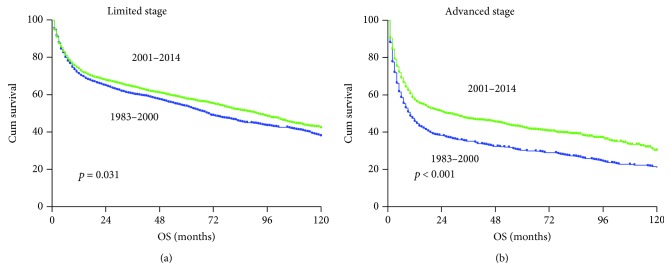
Changes in overall survival for PG-DLBCL patients according to disease stage. The blue and green color curves indicate patients diagnosed in the 1973–2000 era and 2001–2014 era, respectively.

**Figure 6:**
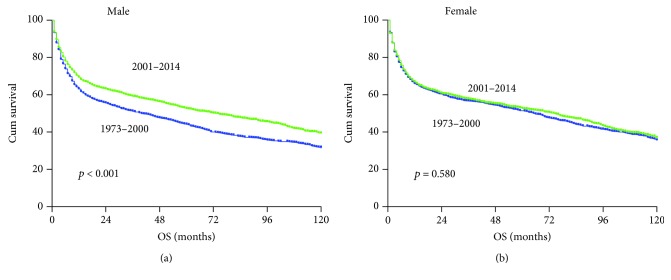
Changes in overall survival for PG-DLBCL patients in two gender groups. The blue and green color curves indicate patients diagnosed in the 1973–2000 era and 2001–2014 era, respectively.

Assessment of the patient survival revealed a trend of improvement across all races in the 2001–2014 era (Figure 4), although in some race group, such improvement did not reach statistical significance due to small number of cases. The overall outcomes for Black race patients remained relatively poor compared to other races, even within the recent era. Survival improvement was observed in male patients with limited and advanced-stage diseases (Figure 5). Stage remains intrinsically associated with survival, even within the most recent era. Individuals with limited stage disease had a lower risk of death than patients with advanced stage. Interestingly, the improvement in overall survival was observed mainly in male patients (Figure 6).

### 3.3. Univariate and Multivariable Analysis

Univariate analysis identified disease diagnosed in the recent era (2001–2014), younger age, and limited stage were associated with better survival, and Black race was associated with poor survival (Table 2). On multivariate Cox regression (Table 2), patients diagnosed in the recent era had lower risk of death compared with that in the previous era (2001–2014 era versus 1973–2000 era: HR = 0.892 (0.836 to 0.952)). Patients older than 60 years, Black race, advanced stage, and males were associated with inferior OS (Table 2). These results were consistent with that of univariate analysis.

## 4. Discussion

The treatment modalities for PG-DLBCL have shifted from surgery as a mainstay of therapy to a more conservative approach using systemic immunochemotherapy with or without radiotherapy in the recent decades [1, 3, 6, 7, 9]. The efficacy of current gastric conservation therapeutic approach is equal to or superior to that of gastric resection. In this population-based study, we reported for the first time that across the 2 eras, the 5-year overall survival have improved from 47% in the 1973–2000 era to 53% in the 2001–2014 era for the whole population of PG-DLBCL patients registered in the SEER database. This improvement was most significant in the White race, younger age, and male patients.

The improvement in OS in PG-DLBCL patients may be attributed to the new treatment modalities and improved supportive care. The nonrandomized trial by the German Multicenter Study Group showed that the 5-year survival rate was comparable between nonsurgical group and surgery group in patients with extranodal DLBCL [1]. As such, gastrectomy for PG-DLBCL is not recommended except for emergencies such as severe bleeding or perforation. Currently, radiation consolidation followed by chemotherapy with or without rituximab for early-stage disease and systemic is accepted by most of clinicians. The addition of rituximab to chemotherapy regimen such as CHOP has been confirmed to significantly improve the overall survival in patients with aggressive B-cell NHL [10–14]. However, whether the therapy containing rituximab can translate a survival benefit in PG-DLBCL remains controversial. Olszewski et al. reported that adding rituximab into CHOP could improve the survival of older patients with extranodal DLBCL and reduce lymphoma-related death for DLBCL of gastrointestinal tract [15]. However, Sohn et al. found that addition of rituximab did not have an impact on the outcomes of patients with PG-DLBCL [16]. Jang et al. also reported that adding rituximab to CHOP regimen yields no benefit in patients with primary extranodal DLBCL, although the specific extranodal sites were not specified [17].

One important finding in our study is the significant improvement in survival in patients under 60 years. The most likely reason is that young patients have better chance to receive intense chemotherapy and new drug treatment. However, the overall outcomes for patients under 60 years remain unsatisfactory. Even in the recent era, the 5-year overall survival was only 68%. Unfortunately, the outcomes for patients aged 60 and older was far inferior, with only 42% survival 5 years after diagnosis in the 2001–2014 era.

Race disparities in patients with DLBCL have been reported previously [18]. Our study showed worse outcomes in patients of the Black race compared with White and other races. The possible explanations could be that Black patients with DLBCL were more likely to present with unfavorable prognostic factors at diagnosis: advanced-stage disease, B symptoms, and extranodal sites [18, 19]. Lower treatment rate and delayed onset of treatment could also contribute to poor clinical outcome [18, 20].

With respect to gender, male patients showed a significant survival improvement. The underlying mechanism was largely unknown. One possible explanation could be the lifestyle changes such as smoking cessation.

Stage is an independent prognostic factor of DLBCL. However, it is worth noting that the 5-year overall survival in the SEER database was only about 56% for early stage patients in the recent era, which is lower than that reported in clinical trials of single-institution studies. In the Southwest Oncology Group (SWOG) randomized phase III trial, the 5-year survival was 82% for early stage patients treated with 3 cycles of CHOP in combination with RT and72% for patients received 8 cycles of CHOP [21]. A Japanese phase II study that evaluated nonsurgical treatment for early stage PG-DLBCL showed a 2-year OS of 94% [22]. In the rituximab era, Tanaka et al. reported a 3-year OS of 90% for patients with localized disease and 64% for patients with advanced stage [23]. The possible factors contributing to the discrepancy between results of SEER analysis and single-institution studies include the intrinsic shortcomings associated with SEER analysis. Information regarding specific treatment regimen and the time course of treatment is not available in the SEER database. This makes it difficult to determine what proportion of PG-DLBCL patients actually received rituximab and other chemotherapy regimens. The lack of centralized pathology review and centralized imaging review could lead to uniformed pathological diagnosis and staging of patients in different registries of SEER-18. These limitations likely contribute to the discrepancy described above.

Nevertheless, the SEER data with over 7000 cases of PG-DLBCL patients shows that stage and age remain as strong prognostic factors. Racial disparities in outcome still exist. Although improvement in survival across the two eras was observed, older patients still had poor prognosis. Despite the significant improvement in survival in the recent era, the overall survival remains unsatisfactory for PG-DLBCL patients. More effective therapeutic approaches are needed to further improve the clinical outcome of PG-DLBCL patients.

## Figures and Tables

**Table 1:** Clinical features of patients with primary gastric DLBCL in the indicated eras.

Clinical features	Era			*p*
	1973–2014 (*N* = 7051)	1973–2000 (*n* = 2865; 40.6%)	2001–2014 (*n* = 4186; 59.4%)	
Median age at diagnosis, years (range)	70 (0–105)	70 (4–100)	71 (0–105)	
Sex *N* (%)				0.004
Male	3944 (55.9%)	1547 (54.0%)	2397 (57.3%)	
Female	3107 (44.1%)	1318 (46.0%)	1789 (42.7%)	
Age *N* (%)				0.288
<60	1859 (26.4%)	766 (26.7%)	1093 (26.1%)	
≥60	5192 (73.6%)	2099 (73.3%)	3093 (73.9%)	
Stage *N* (%)				<0.001^#^
Early	4184 (59.3%)	1615 (56.4%)	2569 (61.4%)	
Advanced	1962 (27.8%)	631 (22.0%)	1331 (31.8%)	
Unknown	905 (12.8%)	619 (21.6%)	286 (6.8%)	
Race *N* (%)				0.018^#^
White	5692 (80.7%)	2367 (82.6%)	3325 (79.4%)	
Black	528 (7.5%)	196 (6.8%)	332 (7.9%)	
Other	793 (11.2%)	297 (10.4%)	496 (11.8%)	
Unknown	38 (0.5)	5 (0.2%)	33 (0.8%)	

^#^Analysis excludes unknown or missing values.

**Table 2:** Univariate and multivariate analysis of clinical parameters associated with overall survival in primary gastric DLBCL.

Variable	Univariate analysis		Multivariate analysis	
	Hazard ratio (95% CI)	*p*	Hazard ratio (95% CI)	*p*
Year of diagnosis				
1973–2000	Reference		Reference	
2001–2014	0.926 (0.868 to 0.988)	0.020	0.892 (0.836 to 0.952)	0.001
Age, years				
<60	Reference		Reference	
≥60	2.249 (2.076 to 2.437)	<0.001	2.383 (2.196 to 2.585)	<0.001
Race				
Black	Reference		Reference	
White	0.922 (0.823to 1.032)	0.158	0.796 (0.710 to 0.892)	<0.001
Other	0.725 (0.627to 0.838)	<0.001	0.662 (0.572 to 0.765)	<0.001
Stage				
Early stage	Reference		Reference	
Advanced stage	1.571 (1.473 to 1.676)	<0.001	1.666 (1.561 to 1.778)	<0.001
Sex				
Male	Reference		Reference	
Female	1.004 (0.944 to 1.068)	0.894	0.931 (0.875 to 0.991)	0.024

## Data Availability

The datasets analyzed during the current study are available in the SEER repository and can be obtained from: https://seer.cancer.gov.
